# Reactivation of BCG vaccination scars after vaccination with mRNA-Covid-vaccines: two case reports

**DOI:** 10.1186/s12879-021-06949-0

**Published:** 2021-12-20

**Authors:** Libin Mohamed, Anne Marie Rosendahl Madsen, Frederik Schaltz-Buchholzer, Anne Ostenfeld, Mihai G. Netea, Christine Stabell Benn, Poul-Erik Kofoed

**Affiliations:** 1 Department of Paediatrics and Adolescent Medicine, Lillebaelt Hospital, University Hospital of Southern Denmark, Sygehusvej 24, 6000 Kolding, Denmark; 2 Bandim Health Project, OPEN, Department of Clinical Research, University of Southern Denmark and Odense University Hospital, 5000 Odense, Denmark; 3 Department of Gynaecology and Obstetrics, Nordsjaellands Hospital, Dyrehavevej 29, 3400 Hilleroed, Denmark; 4 Department of Internal Medicine and Radboud Center for Infectious Diseases, Radboud University Medical Center, Nijmegen, the Netherlands; 5 Department of Immunology and Metabolism, Life and Medical Sciences Institute, University of Bonn, Bonn, Germany; 6 Danish Institute of Advanced Science, University of Southern Denmark, 5230 Odense, Denmark

**Keywords:** BCG, mRNA Covid-19 vaccination, Cross-reaction, Case report

## Abstract

**Background:**

From May 2020 to January 2021, we enrolled 1233 health care workers (HCW) from Danish Hospitals in a randomized trial evaluating whether Bacille Calmette-Guérin (BCG) provides protection against COVID-19. Participants were randomized 1:1 to BCG vs saline and followed for 6 months. From December 2020, Covid-19 vaccines were offered to the HCW. In most cases, BCG vaccination results in a characteristic scar. Reactivation of the BCG scar has been described in children during viral infections and following influenza vaccination, but is mostly associated to Kawasaki’s disease, a disease entity with pathogenesis likely similar to the child Covid-19 complication MIS-C: Multi-System Inflammatory Syndrome. Reactivation of scars after neonatal BCG vaccination has recently been described in four women after Covid-19 mRNA vaccination. Two of our trial participants experienced reactivation of their novel BCG scars after receiving mRNA Covid-19 vaccination 6 to 8 months post-BCG.

**Case presentations:**

Two female HCW participants that had been randomly allocated to BCG in the BCG-DENMARK-COVID trial, spontaneously reported itching and secretion at the BCG scar site after having received mRNA Covid-19 vaccination (Moderna and Pfizer-BioNTech) 6 to 8 months following inclusion and BCG vaccination. One participant, who had a larger BCG skin reaction, noticed re-appearing symptoms after both the first and the second COVID-vaccine dose, while the other participant only noted symptoms after the second dose. Both had been BCG vaccinated during childhood, and no reactivation was noted in the older scars. No treatment was needed or provided.

**Conclusions:**

The reactivation of the BCG scar after receiving mRNA vaccine might have been caused by cross-reactivity between BCG and SARS-CoV-2. In both cases, the symptoms were bothersome, but self-limiting and left no sequelae. The risk of reactivation at the scar site is thus not a reason to avoid vaccination with either vaccine.

## Background

Vaccines are designed to provide protection against a specific microorganism, inducing protective immunity against a specific disease. A series of observational studies and randomized controlled trials indicate that in addition, some vaccines can induce beneficial non-specific effects, enhancing the capacity of the immune system to handle heterologous pathogens. Clinical trials are ongoing to test if vaccines with known beneficial non-specific effects could serve as stop-gap vaccines against Coronavirus disease 2019 (Covid-19). One of the vaccines being investigated is Bacille Calmette-Guérin (BCG), a live-attenuated strain of *Mycobacterium bovis* used against tuberculosis (TB).

In most countries where tuberculosis is still a public health issue, BCG is recommended to all neonates at birth or as soon thereafter as possible. In countries with low incidence of TB, as is the case for Denmark, BCG vaccine is only given to high-risk groups. Up to 97% of people who receive BCG vaccine experience a skin reaction at the injection site 2 to 4 weeks after vaccination [1, 2]. The reaction heals within 2 to 5 months. In most cases the vaccination leaves a scar with a diameter of approximately five mm. Observational studies and randomized controlled trials indicate that BCG at birth is associated with a 30% to 50% reduction in neonatal mortality [3, 4]. BCG induces protection against infections such as sepsis and respiratory infections [4, 5].

Based on these findings, more than 20 trials are currently ongoing worldwide to test if BCG vaccination can provide full or partial protection against getting Covid-19. One of these is the BCG-DENMARK-COVID trial (NCT04373291), which from May 2020 to January 2021 enrolled 1233 health care workers (HCW) at nine Danish hospitals [6]. Participants were randomized 1:1 to standard dose BCG-Denmark or saline by intracutaneous administration in the right deltoid region to distinguish any reaction or scar formation from previous BCG immunization, which is traditionally administered in the left deltoid region. The participants were followed for 6 months with weekly questionnaires about symptoms and work absenteeism.

Specific COVID-19 vaccines were developed with unprecedented speed and rolled out to the HCW employed at Danish hospitals from December 27, 2020. Hence, many participants received or will receive Covid-19 vaccines during follow-up. Two participating HCW randomized to receive BCG reported reoccurring symptoms from the BCG scar site after receiving mRNA Covid-19 vaccines.

## Case presentations

### Case 1

A 53-year-old female participant in the BCG-DENMARK-COVID trial was included in early June 2020 and randomized to BCG, which was applied intradermally in the right deltoid region. A rather strong local skin reaction to the vaccine followed, with clear, yellowish serous secretion from the injection site lasting 4 to 5 months. Additionally, swollen and sore lymph nodes in the axil on the vaccinated side were noted. The lymph node symptoms led to her being examined for breast cancer in January 2021. The participant had been BCG-vaccinated as a child at school entry, and a scar from the childhood vaccination was noted on her right shoulder at the trial inclusion procedure. According to her mother, she had also reacted strongly to the childhood BCG vaccination. As far as she (and her mother) knows, she has never been exposed to TB. The participant is healthy and takes no medication.

By the end of January 2021, she received the first Moderna Covid-19 vaccination in the left arm. She received no other vaccines during follow-up. The participant reacted to the Covid-19 vaccine with fever, muscle pain, and a large local reaction (the area being red and inflamed) which subsided within a few days. One to two days after vaccination, the trial BCG vaccination site scar began to itch and she experienced renewed secretion from the site, and the lymph nodes felt sorer. The itching and the secretion lasted for a week.

Three weeks later, she received the second Moderna vaccination, after which she felt ill again. Also, this time the BCG site began to itch during the following day. There were no other symptoms, and the secretion from the BCG scar did not reappear. The itching lasted for 2 weeks. No treatment was needed or provided. Again, she did not notice symptoms from the childhood BCG scar.

### Case 2

A 49-year-old female participant was enrolled and randomized to BCG in June 2020. The vaccine was applied intradermally in the right deltoid region. She was not aware of having received a BCG vaccination as a child. However, when she was included in the trial, a BCG scar was noted on the right arm. In October 2020, she received an influenza vaccination (Vaxigriptetra®). No reaction at the trial BCG scar was noted. The participant takes omeprazole daily as a treatment for her gastroesophageal reflux disease and levocetirizin (Xyzal®) for her chronic urticaria.

Late December 2020, she received the Pfizer-BioNTech Covid-19 vaccine in the left arm with no subsequent reaction at any of the BCG scar sites. After her second dose of Pfizer-BioNTech vaccine late January 2021, also in the left arm, she noted itching, clear yellowish secretion, and some bleeding from the BCG scar site on the right arm. She did not experience swollen lymph nodes. The symptoms lasted for 2 weeks and resolved without treatment. There was no reaction at the site of the childhood BCG scar. She had no other symptoms after the Pfizer-BioNTech vaccine.

## Discussion and conclusion

After receiving mRNA Covid-19 vaccines, two HCW who had recently been vaccinated with BCG as a part of the BCG-DENMARK-COVID trial experienced reactivation at the site of the trial BCG vaccines administered 6 to 8 months earlier. There was no reactivation in childhood BCG scars. The participant who reacted most strongly after receiving BCG vaccination both as a child and when entering the BCG-DENMARK-COVID trial experienced symptoms from the trial BCG scar site already after the first Covid-19 vaccination, whereas the participant who had only had moderate symptoms following the BCG inoculation 6 months earlier only noted reactivation after the second injection.

Reactivation of BCG scars has previously been reported following influenza vaccination [7, 8], but the participant who received an influenza vaccination 2 months before the Covid-19 vaccination did not experience any such reactions.

Two recent case reports described similar reactions in scars after neonatal BCG vaccines in four health care workers aged between 28- and 45-years following mRNA Covid-19 vaccines [9, 10]. Reactivation of BCG scars has also been described during viral infections in children, e.g., measles and human herpes virus type 6 [11–13]. However, reactivation of BCG scars has mainly been associated with Kawasaki disease (KD), and local reactions such as erythema and induration at the BCG scar site have even been suggested as a diagnostic tool in diagnosing KD [14]. A study from Singapore, where BCG is given to all new-born infants, reported that 43% of patients with KD developed reactivation at the BCG scar site, most frequently the youngest children [13]. The study indicated that the higher prevalence in the youngest children was due to the shorter duration between BCG inoculation and onset of KD, rather than age per se [13]. This could explain why we only observed reactivation in the most recent BCG scars.

Interestingly, a new pediatric disease associated with Covid-19, called multisystem inflammatory syndrome in children (MIS-C) has been identified, with pathogenesis and clinical presentation similar to KD [15]. The fact that COVID-19 can cause MIS-C, that MIS-C resembles KD, and KD is associated with BCG reactivation points to some degree of shared etiology. The pathophysiology of MIS-C is still unknown [15]. MIS-C has mostly been diagnosed in high-income countries where BCG is not widely used, and to our knowledge, no reactivations of BCG scars have been reported in children suffering from MIS-C. However, this is likely due to the rarity of MIS-C, and the low epidemiological chance that this would happen in a child vaccinated with BCG. On the other hand, based on the available evidence, BCG scar reactivation in children with MIS-C could be anticipated in countries where BCG is used routinely.

Live BCG organisms have been shown to remain in BCG-vaccinated mice for up to 5 months [16], and have been cultured from children born to HIV positive mothers 9 years after receiving BCG at birth [17]. It is, however, improbable that the changes seen in the two cases are due to live BCG bacteria. It is thus more likely that the local reaction was induced by cross-reactivity between BCG microbial components persisting at the site of vaccination and SARS-CoV-2 vaccines. An in silico analysis suggested that BCG vaccine has the potential to generate cross-reactive T cells against SARS-CoV-2 [18]. Corroborating this finding, eight BCG-derived peptides with significant sequence homology to either SARS-CoV-2 nonstructural protein 3 (NSP3) or nonstructural protein 13 (NSP13) derived peptides were recently identified [19]. Interestingly, human CD4+ and CD8+ T cells primed with a BCG-derived peptide developed enhanced reactivity to its corresponding SARS-CoV-2 derived peptide, supporting the hypothesis that BCG vaccination induces cross-reactive SARS-CoV-2 specific T cell responses [19]. Furthermore, a high homology of the SARS-CoV-2 envelope protein with the consensus protein LytR C unique to *Mycobacteria* indicates that BCG vaccination induces a specific immunity against SARS CoV-2 that targets the viral envelope protein that is essential for infectivity [20]. The reactivation of the BCG scar experienced by two HCW after receiving mRNA vaccines might therefore have been caused by an immunological reaction due to the cross reactivity between BCG and SARS-CoV-2.

In the BCG-DENMARK-COVID trial, 614 HCW participants were randomized to receive BCG. Two participants reported BCG scar reactivation following Covid-19-vaccination. In both cases, the symptoms were irritating, but self-limiting, and they left no sequelae. Both participants were glad to have contributed to the trial and would be happy to participate in similar trials in the future. Our case one participant felt that her general health had improved compared to before the trial, whereas the participant from case two felt her general health to be unchanged.

A limitation of the cases reported is that no testing was done to elucidate the pathogenesis causing the symptoms. However, the two participants experienced the symptoms to the degree that they spontaneously reported them, why it is important for clinicians to know about the possibility of seeing similar reactions and it also seems worthwhile to alert physicians to the fact that BCG scar reactivation might be an indicator of MIS-C. Still, there is no reason to avoid vaccination with either of the mRNA Covid-19 vaccines.

## Data Availability

All data are contained in the manuscript.

## Abbreviations

BCG: Bacillus Calmette-Guérin
Covid-19: Coronavirus disease 2019
HCW: Health care workers
KD: Kawasaki disease
MIS-C: Multisystem inflammatory syndrome in children
TB: Tuberculosis
